# Prenatal alcohol exposure and offspring cognition and school performance. A ‘Mendelian randomization’ natural experiment

**DOI:** 10.1093/ije/dyt172

**Published:** 2013-09-24

**Authors:** Luisa Zuccolo, Sarah J Lewis, George Davey Smith, Kapil Sayal, Elizabeth S Draper, Robert Fraser, Margaret Barrow, Rosa Alati, Sue Ring, John Macleod, Jean Golding, Jon Heron, Ron Gray

**Affiliations:** ^1^MRC Integrative Epidemiology Unit, University of Bristol, Bristol, UK, ^2^School of Social and Community Medicine, University of Bristol, Bristol, UK, ^3^Section of Developmental Psychiatry, University of Nottingham, Nottingham, UK, ^4^Department of Health Sciences, University of Leicester, Leicester, UK, ^5^School of Medicine, University of Sheffield, Sheffield, UK, ^6^Clinical Genetics, University Hospitals of Leicester, Leicester, UK, ^7^School of Population Health, University of Queensland, Brisbane, Queensland, Australia and ^8^National Perinatal Epidemiology Unit, University of Oxford, Oxford, UK

**Keywords:** Alcohol dehydrogenase, causality, cognition, confounding factors, educational measurement, ethanol, Mendelian randomization analysis, pregnancy

## Abstract

**Background** There is substantial debate as to whether moderate alcohol use during pregnancy could have subtle but important effects on offspring, by impairing later cognitive function and thus school performance. The authors aimed to investigate the unconfounded effect of moderately increased prenatal alcohol exposure on cognitive/educational performance.

**Methods** We used mother-offspring pairs participating in the Avon Longitudinal Study of Parents and Children (ALSPAC) and performed both conventional observational analyses and Mendelian randomization using an *ADH1B* variant (rs1229984) associated with reduced alcohol consumption. Women of White European origin with genotype and self-reported prenatal alcohol consumption, whose offspring’s IQ score had been assessed in clinic (*N* = 4061 pairs) or Key Stage 2 (KS2) academic achievement score was available through linkage to the National Pupil Database (*N* = 6268), contributed to the analyses.

**Results** Women reporting moderate drinking before and during early pregnancy were relatively affluent compared with women reporting lighter drinking, and their children had higher KS2 and IQ scores. In contrast, children whose mothers’ genotype predisposes to lower consumption or abstinence during early pregnancy had higher KS2 scores (mean difference +1.7, 95% confidence interval +0.4, +3.0) than children of mothers whose genotype predisposed to heavier drinking, after adjustment for population stratification.

**Conclusions** Better offspring cognitive/educational outcomes observed in association with prenatal alcohol exposure presumably reflected residual confounding by factors associated with social position and maternal education. The unconfounded Mendelian randomization estimates suggest a small but potentially important detrimental effect of small increases in prenatal alcohol exposure, at least on educational outcomes.

## Introduction

Heavy alcohol drinking during pregnancy is known to have detrimental effects on the foetus.^1–4^ Whereas the majority of expectant mothers do not drink heavily, there is concern that the more prevalent light-to-moderate alcohol use could have subtle but important effects on offspring, by influencing cognitive function and thus school performance.^5–8^ Such an effect could be of substantial public health importance, as educational success is a key influence on subsequent life trajectories.

Epidemiological research on the effects of alcohol use during pregnancy has generated contrasting and occasionally paradoxical findings—reminding us that identifying true causal effects in observational research is often difficult.^9^ In recent analyses based on British, Australian and Danish cohorts examining effects on children’s cognitive development, attention, motor function and risk of behavioural problems, light-to-moderate alcohol consumption and occasional binge drinking in pregnancy appeared to be associated mostly with no change but occasionally with modest, non-systematic improvements.^10–16^ However, a recent review of systematic reviews on this topic concluded that the results of studies showing significant (protective) effects from low levels of prenatal alcohol exposure are most likely explained by bias due to confounding and/or misclassification of exposure or outcome.^17^

We believe that in studies of low-to-moderate alcohol consumption during pregnancy, residual confounding by factors more prevalent among light and moderate alcohol consumers, such as relative affluence, could potentially obscure detrimental effects if they existed, biasing results in the opposite direction. Children of affluent parents typically enjoy better outcomes across a range of measures, especially educational outcomes. This apparent advantage may arise despite, rather than because of, their mothers’ alcohol use. So although prenatal alcohol exposure may be harmful at any level, it is possible that it could be so confounded that light and moderate levels of consumption show either no effect or even beneficial effects. We view this as an important limitation of the interpretability of observational studies in this area, in line with the critique by O’Leary and Bower.^17^

Where experimental studies are unfeasible, the quasi-experimental approach of ‘Mendelian randomization’,^18^ involving the use of genetic variants influencing the environmental exposure of interest, offers an alternative. Genotype-outcome associations give an unconfounded ‘intention to treat’ estimate of effects of that exposure (providing the assumptions are met), since genotype is randomly assigned at meiosis and unrelated to factors that confound conventional observational associations.^18^^,^^19^ This is true irrespective of the magnitude of the influence of genotype on the exposure, relative to other influences on the exposure and outcome. We successfully used this approach in a recent paper showing that 4 out of an initial set of 10 candidate variants in offspring alcohol metabolixing genes were associated with their cognitive ability at age 8 years in the population-based Avon Longitudinal Study of Parents and Children (ALSPAC).^20^ This was only seen among those whose mothers reported drinking alcohol at most in moderation during pregnancy, *in utero* exposure being the only plausible alcohol exposure for these children.

Another crucial aspect is timing of the exposure, with misclassification likely to reflect some of the inconsistencies in previous studies.^17^ Prenatal alcohol exposure is most likely to affect neuro-developmental outcomes if it happens early in pregnancy,^21^ with the critical period possibly preceding recognition of pregnancy or even conception.^22^ However, some studies considered alcohol exposure at any time-point in pregnancy.^23^ For this reason, we investigated the association of early prenatal alcohol exposure based on mothers’ self-reported alcohol use before and in the early stages of pregnancy (during the first gestational trimester) with child intelligence quotient (IQ) at age 8 years and school performance at age 11 years among the offspring of women participating in ALSPAC. We also used a Mendelian randomization approach and investigated associations between the children’s outcomes and a maternal variant in an alcohol dehydrogenase gene (*ADH1B*) predicting lower alcohol consumption before and during early pregnancy.^24^ Carriers of the rare allele (faster metabolizers^25^) had been shown to be more likely to abstain in the first trimester and consume less alcohol before pregnancy, and less likely to ‘binge drink’ during pregnancy.^24^ Based on current evidence, this maternal *ADH1B* genotype is the most robust genetic instrument for prenatal alcohol exposure, being linked with differences in both alcohol consumption and peak blood levels. This choice of variant is particularly good for exposure to alcohol in early pregnancy before the offspring genes start being expressed, and this different focus, as well as the inclusion of school results outcomes, makes this new study complementary to but distinct from our earlier work.^20^ Our hypothesis was that a causal relationship between maternal alcohol consumption in early pregnancy and offspring cognitive and educational outcomes should be consistently reflected across both conventional observational and Mendelian randomization analyses, whereas discrepant findings would suggest residual confounding in the former.

## Materials and methods

### Participants

ALSPAC^26^^,^^27^ is a population-based longitudinal study that recruited 14 541 pregnant women living around the city of Bristol, South West England (UK), with expected delivery date between 1 April 1991 and 31 December 1992. Details are available on the study website http://www.bristol.ac.uk/alspac/.

### Measurement of alcohol intake

ALSPAC mothers completed a questionnaire at 18 weeks of gestation, including questions on the amount of alcohol consumed on average before the current pregnancy and during the first trimester. One drink was specified as one UK unit (8 g) of alcohol, and women were asked to recall their frequency of drinking as 0 units/week, <1 unit/week, ≥1 unit/week, 1–2 units/day, 3–9 units/day or 10+ units/day. A similar questionnaire asked about consumption 8 months post-delivery. For all exposure periods, the three highest categories were grouped together because of small numbers. Information on binge drinking was derived from further questions asking about ever consuming 4+ alcoholic drinks in one occasion.

### Measurement of cognition and school performance

The study outcomes were age-standardized IQ scores (clinic-based testing at age 8 years using a shortened version of the Wechsler Intelligence Scale for Children: WISC-III^28^) and age-standardized National Curriculum Key Stage 2 (KS2) scores (assessment at 11 years of age by examinations in English, Mathematics and Science^29^ for children at non-fee-paying schools). The distribution of the global scores were similar, with mean around 100 and standard deviation (SD) around 15. IQ’s arithmetic component and KS2’s mathematics component were used in sensitivity analyses, both expressed as standardized scores (mean 100, SD 15) so that effect estimates for these are comparable to those for overall IQ and KS2.

### Measurement of potential confounders

The following were derived based on data abstracted from mother-completed questionnaires: maternal age at delivery, parity, family social class (highest social class of mother or partner, based on occupation and determined according to the 1991 British Office of Population Statistics classification^30^), maternal education (based on O level, an exam-based qualification for students aged 14–16 years^31^), smoking during first trimester of pregnancy, maternal diet in third trimester (weekly intake of folate, calcium, iron and vitamin C, calculated from food frequency questionnaires), maternal Edinburgh Postnatal Depression Scale (EPDS, which may be used to assess both antenatal and postnatal depressive symptoms^32^).

### Genotyping

The *ADH1B* polymorphism rs1229984 was genotyped by KBioscience using the KASPar chemistry (http://www.kbioscience.co.uk/genotyping/genotyping-chemistry.htm). Blind duplicates, plate-identifying repeat samples and Hardy–Weinberg equilibrium tests were used as quality control checks.^24^

### Ethics approval

Ethical approval came from the ALSPAC Ethics and Law Committee (IRB 00003312) and Local Research Ethics Committees (LREC).

### Statistical analysis

We used univariate linear regression models to examine the relationships between alcohol intake before and in the first trimester of pregnancy and child IQ and KS2 scores. These models were then adjusted for potential confounders, identified as maternal factors previously associated with poor offspring developmental outcomes, which also showed high correlation with maternal alcohol use in pregnancy in this study.

As the minor allele frequency for rs1229984 was 2.4%, we grouped rare homozygotes (0.04%) and heterozygotes (4.66%) together and assumed a dominant effect, since our previous analysis suggested this was appropriate.^24^

**Figure 1:**
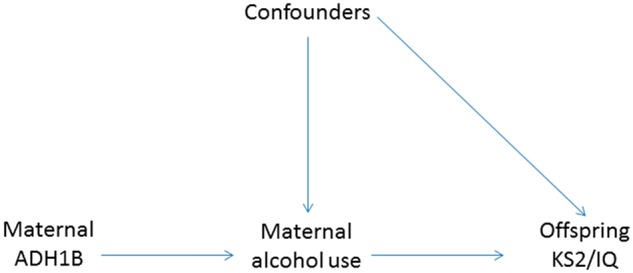
Mendelian randomization model assumptions. 1: the *ADH1B* genotype (rs1229984) is associated with maternal alcohol consumption. 2: there is no association between the genotype and confounding factors. 3: the genotype does not affect the outcome by any path other than maternal alcohol consumption. By testing the maternal *ADH1B*-offspring outcome association, we are testing the association between maternal alcohol use and offspring outcomes, provided that the assumptions hold

The main assumptions underlying Mendelian randomization, illustrated in Figure 1, are 1: the genotype (rs1229984) is robustly associated with maternal alcohol consumption, 2: there is no association between the genotype and confounding factors and 3: the genotype does not affect the outcome by any path other than maternal alcohol consumption (i.e. there is no pleiotropy). To test the association between rs1229984 and maternal alcohol consumption before, during and after pregnancy we used chi-square tests (assumption 1). An empirical check of assumption 2 was attempted by testing the association of a number of measured potentially confounding factors with maternal rs1229984 genotype. These were then compared with the associations between the same potential confounders and maternal alcohol consumption. Assumption 3 cannot be tested unless multiple genetic variants are available to use as instrumental variables.

To test for the presence of a causal effect of alcohol on the outcomes in the context of Mendelian randomization, we tested the association between rs1229984 and IQ and KS2 scores using linear regression. Given the negative association between the rare allele and alcohol consumption, a genetic effect in the positive direction would indicate a detrimental effect of alcohol on the scores (women drinking less have children who do better at the tests), and conversely one in the negative direction would imply a beneficial effect of alcohol. To avoid population stratification,^24^ models were restricted to women reporting White European origin and adjusted for ten ancestry-informative principal components derived from analysis of genome-wide association study panel data.^33^

We formally tested for genotype X alcohol interaction, in models assuming a linear trend across categories of alcohol drinking (first trimester). Our prior hypothesis for doing this was that the genetic effect would increase for increasing levels of alcohol consumption. This is because the more mothers drink, the larger the potential for differences in cumulative exposure to ethanol to the developing foetus according to maternal genotype, with offspring of mothers carrying the rare allele being exposed to less ethanol due to reduced consumption and faster ethanol clearance, compared with offspring of common allele carriers.

In sensitivity analyses, we estimated the effect of *ADH1B* genotype on both outcomes after excluding women who reported binge drinking or drinking 7+ units/week during pregnancy. We also considered the relationship between child’s genotype and KS2 scores to check that the effect was due to maternal rather than child’s genotype. Additionally, to check whether number processing domains were particularly affected by prenatal alcohol exposure, as previously suggested,^34^ we examined IQ’s arithmetic component and KS2’s mathematics component.

A complete case analysis was performed for exposures-outcomes and exposures-potentially confounding factors combinations. Measurement of IQ was conditional on attending a clinic visit, and therefore prone to loss to follow-up bias, whereas KS2 scores available from routine school assessments were available for most children, and only missing for a small number of pupils at fee-paying schools. To gauge the possible extent of selection bias due to loss to follow-up, we repeated both the observational (self-reported alcohol consumption as exposure) and genetic (using the *ADH1B* variant as exposure) analyses on KS2 scores restricted to mother-child pairs with available IQ data.

All statistical tests were 2-sided. Analyses were conducted using Stata 12.

## Results

We identified 12 075 mother-child pairs, excluding twins and second or later ALSPAC births, children dying before their first birthday and women of non-White ethnicity. Of these, 11 562 mothers reported pre-pregnancy and first-trimester alcohol consumption, of whom 7084 were successfully genotyped for rs1229984. For analyses investigating the association of maternal alcohol consumption with child KS2 and IQ scores we used 8530 and 5711 mother-child pairs, respectively, and for the Mendelian randomization analyses we used 6268 and 4061 mother-child pairs, respectively.

**Figure 2:**
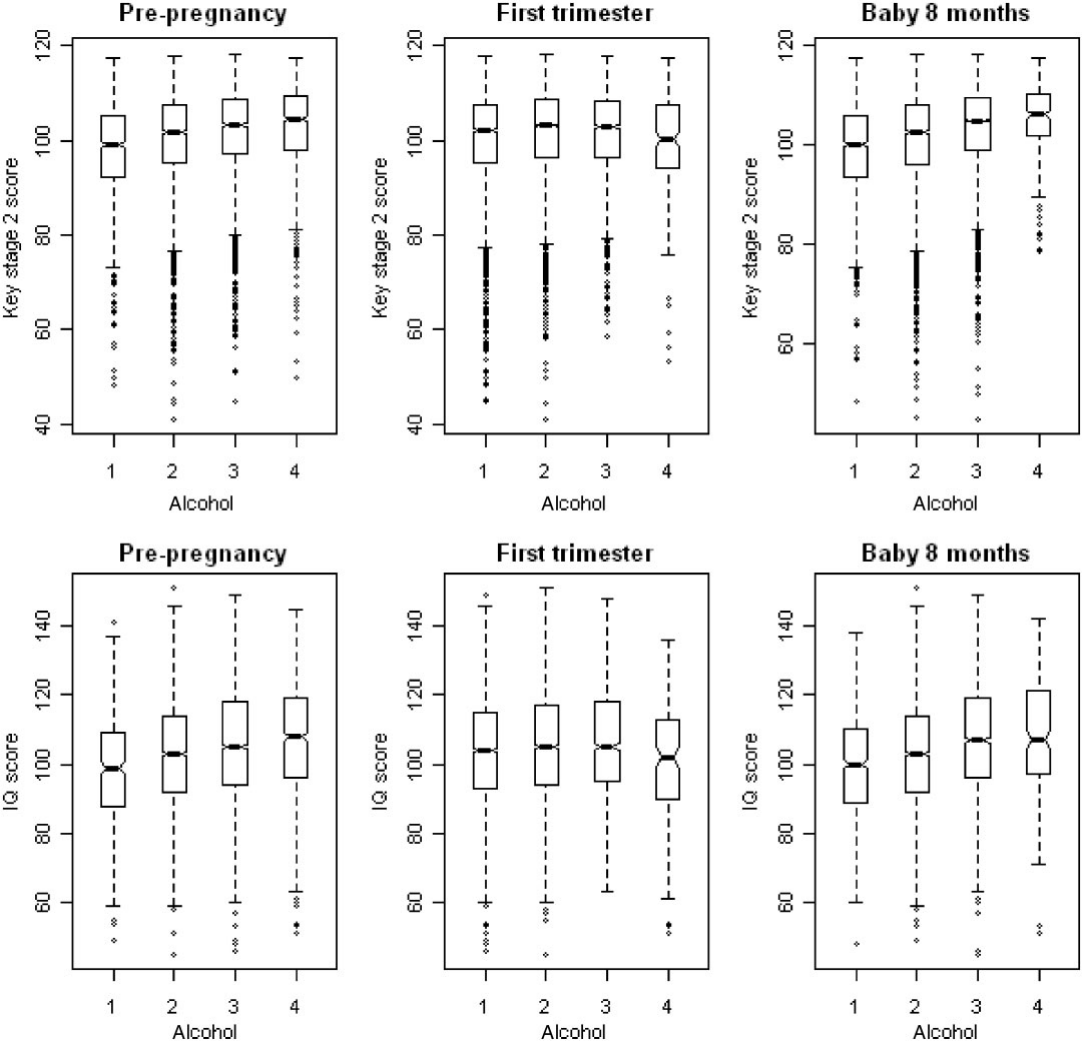
Box plots showing the distribution of children’s IQ at age 8 years and Key Stage 2 scores at age 11 years by levels of maternal self-reported alcohol consumption before, during pregnancy (in first trimester) and after pregnancy (8 months post-delivery). Avon Longitudinal Study of Parents and Children, 1991–92. Alcohol consumption categories 1: 0 units/week, 2: <1 unit per week, 3: 1–6 units per week, 4: 7+ units per week

There was a strong relationship between alcohol consumption before pregnancy and child KS2 and IQ scores, with heavier drinkers tending to have children with higher scores (Table 1 and Figure 2). Adjustment for measured confounding factors produced an attenuation of effect sizes, but there remained strong and relatively substantial evidence of an effect (Table 1). The patterns of association with mothers’ drinking during the first trimester were in the same direction for moderate use up to 6 units/week (associated with better cognitive outcomes), but heavier use of 7+ units/week suggested a detrimental effect on child IQ scores (Table 1 and Figure 2). Compared with associations with pre-pregnancy drinking, effects were of smaller magnitude, statistically less robust and showed greater attenuation on adjustment for confounders (Table 1).

**Table 1:** Average change in educational/cognitive scores by increasing frequency of alcohol consumption before and during pregnancy (first trimester). Avon Longitudinal Study of Parents and Children, 1991–92

Alcohol consumption		KS2 score at age 11 years			IQ score at age 8 years		
		Mean difference (SE)			Mean difference (SE)		
		*n*	Crude	Adjusted^a^	*n*	Crude	Adjusted^a^
Pre-pregnancy	0 units/week	517	0	0	302	0	0
	<1 unit/week	3295	1.95 (0.42)	0.63 (0.39)	2143	4.13 (0.99)	1.83 (0.93)
	1–6 units/week	3806	3.31 (0.42)	1.01 (0.38)	2580	6.38 (0.98)	2.10 (0.93)
	7+ units/week	912	4.08 (0.49)	1.24 (0.45)	686	8.62 (1.12)	2.99 (1.06)
	*P*^b^		<0.0001	<0.0001		<0.0001	<0.0001
First trimester	0 units/week	3801	0	0	2294	0	0
	<1 unit/week	3425	0.67 (0.21)	0.19 (0.31)	2349	0.94 (0.47)	0.20 (0.44)
	1–6 units/week	1156	0.69 (0.30)	0.28 (0.19)	774	1.46 (0.67)	0.29 (0.63)
	7+ units/week	130	−0.07 (0.79)	0.73 (0.97)	89	−2.20 (1.76)	−3.14 (1.64)
	*P*^b^		0.007	0.132		0.031	0.054

KS2, Key Stage 2; IQ, intelligence quotient; SE, standard error.

^a^Adjusted for family social class and the following maternal characteristics: age, education, parity, smoking during pregnancy, diet (calcium, vitamin C, iron and folate intake), Edinburgh postnatal depression score.

^b^*P*-values for linear trend across categories of alcohol consumption.

Alcohol drinking around the time of pregnancy was strongly associated with socioeconomic position, maternal education and lifestyle factors (Tables 2 and 3). Compared with women who at most drank very little before pregnancy, moderate drinkers were older, more highly educated and of higher social class, and had better diets. Younger, deprived mothers tended to drink less before pregnancy (Table 2). The social patterning of alcohol consumption during the first trimester was somewhat different, with both abstainers and women drinking 7+ units/week coming from seemingly more disadvantaged backgrounds than women drinking up to 6 units/week (Table 3).

**Table 2:** Associations of potentially confounding variables with maternal alcohol intake pre-pregnancy (*n* = 11 386). Avon Longitudinal Study of Parents and Children, 1991–92

	0 units/week (*n* = 804)	<1 unit/week (*n* = 4273)	1–6 units/week (*n* = 5003)	7+ units/week (*n* = 1306)	*P**^c^*
Mother’s age in years (mean, SD)	26.7 (5.0)	27.8 (4.8)	28.3 (4.8)	29.4 (5.3)	<0.001
Parity (1st baby *vs* previous babies)	559 (69.5%)	3230 (75.6%)	4127 (82.5%)	1111 (85.1%)	<0.001
Education (higher *vs* lower)^a^	166 (20.6%)	1,256 (29.4%)	2016 (40.3%)	622 (47.6%)	<0.001
Social class (manual *vs* professional)	201 (25.0%)	885 (20.7%)	753 (15.1%)	160 (12.3%)	<0.001
Smoked in 1st trimester (yes *vs* no)	257 (32.0%)	987 (23.1%)	1175 (23.5%)	444 (34.0%)	<0.001
Calcium mg/week (mean, SD)^b^	6173 (2067)	6480 (1972)	6720 (1918)	6777 (2046)	<0.001
Vitamin C mg/week (mean, SD)^b^	504 (245)	539 (234)	584 (240)	591 (247)	0.55
Iron mg/week (mean, SD)^b^	68.1 (24.7)	71.6 (22.7)	74.8 (22.5)	74.4 (23.3)	<0.001
Folate mg/week (mean, SD)^b^	1639 (548)	1725 (503)	1787 (497)	1761 (502)	<0.001
High EPDS score (more *vs* less depressed)	103 (12.8%)	432 (10.1%)	462 (9.2%)	168 (12.9%)	<0.001

SD, standard deviation; EPDS, Edinburgh Postnatal Depression score.

^a^O level or above, including university degrees.

^b^As estimated from dietary intake, excluding supplements.

^c^*P*-values for linear trend across categories of alcohol consumption.

**Table 3:** Associations of potentially confounding variables with maternal alcohol intake in first trimester of pregnancy (*n* = 11 386). Avon Longitudinal Study of Parents and Children, 1991–92

	0 units/week (*n* = 5106)	<1 unit/week (*n* = 4476)	1–6 units/week (*n* = 1595)	7+ units/week (*n* = 209)	*P*^c^
Mother’s age in years (mean, SD)	27.6 (4.9)	28.5 (4.8)	28.8 (5.1)	29.4 (5.8)	<0.001
Parity (1st baby *vs* previous babies)	4141 (81.1%)	3518 (78.6%)	1220 (76.5%)	160 (76.6%)	<0.001
Education (higher *vs* lower)^a^	1685 (33.0%)	1681 (37.6%)	619 (38.8%)	70 (33.5%)	<0.001
Social class (manual *vs* professional)	968 (19.0%)	729 (16.3%)	281 (17.6%)	46 (22.0%)	<0.001
Smoked in 1st trimester (yes *vs* no)	1041 (20.4%)	1083 (24.2%)	542 (34.0%)	124 (59.3%)	<0.001
Calcium mg/week (mean, SD)^b^	6495 (1985)	6663 (1929)	6759 (1998)	6497 (2103)	<0.001
Vitamin C mg/week (mean, SD)^b^	559 (243)	567 (237)	562 (241)	560 (264)	0.55
Iron mg/week (mean, SD)^b^	72.5 (23.3)	73.7 (22.4)	73.7 (23.0)	68.7 (24.9)	<0.001
Folate mg/week (mean, SD)^b^	1745 (516)	1763 (492)	1749 (505)	1660 (540)	0.02
High EPDS score (more *vs* less depressed)	516 (10.1%)	438 (9.8%)	177 (11.1%)	34 (15.5%)	<0.001

SD, standard deviation; EPDS, Edinburgh postnatal depression score.

^a^O-level or above, including university degrees.

^b^As estimated from dietary intake, excluding supplements.

^c^*P*-values for linear trend across categories of alcohol consumption.

In contrast to the strong associations between measured confounding variables and maternal drinking, there was little evidence of association between the *ADH1B* genotype and potential confounders (Table 4). In addition, we confirmed that women drinking less were more likely to carry the rare allele at rs1229984, particularly for consumption before and during pregnancy (as previously shown^24^), and to a lesser extent post-pregnancy (Supplementary Table S1, available as Supplementary data at *IJE* online), thus validating the single-nucleotide polymorphism (SNP) as our instrumental variable.

**Table 4:** Associations of potentially confounding variables with maternal *ADH1B* genotype (*n* = 7084). Avon Longitudinal Study of Parents and Children, 1991–92

	Rare allele carrier^c^ (*n* = 333)	Non-carrier^c^ (*n* = 6751)	*P*^d^
Mother’s age in years (mean, SD)	28.5 (4.7)	28.2 (4.8)	0.248
Parity (1st baby *vs* previous babies)	153 (45.9%)	3078 (45.6%)	0.945
Education (higher *vs* lower)^a^	130 (39.0%)	2403 (35.6%)	0.189
Social class (manual *vs* professional)	173 (52.0%)	3435 (50.9%)	0.344
Smoked in 1st trimester (yes *vs* no)	70 (21.0%)	1634 (24.2%)	0.094
Calcium mg/week (mean, SD)^b^	6741 (2010)	6555 (1945)	0.106
Vitamin C mg/week (mean, SD)^b^	582 (247)	561 (240)	0.132
Iron mg/week (mean, SD)^b^	74.6 (23.3)	72.6 (22.7)	0.126
Folate mg/week (mean, SD)^b^	1786 (513)	1741 (501)	0.128
High EPDS score (more *vs* less depressed)	57 (17.1%)	1343 (19.9%)	0.841

SD, standard deviation; EPDS ,Edinburgh postnatal depression score.

^a^O level or above, including university degrees.

^b^As estimated from dietary intake, excluding supplements.

^c^Referred to the mother. Carriers of the rare allele on average drank less alcohol.

^d^*P*-values for difference between genotype groups.

For the Mendelian randomization approach, we tested associations between rs1229984 and the outcomes (Table 5). There was evidence of association with KS2 score, with children of mothers carrying the rare allele scoring on average 1.7 (*P* = 0.009) points higher than those of non-carriers, but no evidence of association with IQ (mean difference = −0.01, *P* = 0.979).

**Table 5:** Differences in IQ scores at age 8 years and KS2 scores at age 11 years between *ADH1B* rare allele carriers and non-carriers, stratified by maternal alcohol intake in first trimester. Avon Longitudinal Study of Parents and Children, 1991–92. Models adjusted for ancestry-informative principal components to account for population stratification

Response	Alcohol drinking in 1st trimester	Numbers in analysis		Effect estimate		*P-*value^a^	*P*-value^b^
		Carrier^c^	Non-carrier^c^	Mean difference	95% CI		
IQ score	0 units/week	76	1221	0.4	−3.4, 4.1	0.850	
	<1 unit/week	52	1207	−1.0	−5.5, 3.5	0.899	
	1–6 units/week	10	439	−0.5	−9.1, 8.1	0.976	
	7+ units/week	2	53	12.4	−10.5, 35.2	0.559	
	Overall	140	2915	−0.01	−2.8, 2.7	0.979	
							0.865
KS2 score	0 units/week	113	1952	1.6	−0.1, 3.3	0.071	
	<1 unit/week	67	1781	1.6	−0.6, 3.9	0.070	
	1–6 units/week	17	634	2.3	−2.2, 6.7	0.119	
	7+ units/week	2	89	8.3	−4.5, 21.2	0.087	
	Overall	199	4456	1.7	0.4, 3.0	0.009	
							0.868

KS2, Key Stage 2; IQ, intelligence quotient; CI, confidence interval.

^a^*P*-values from *t* tests for differences of means within each drinking stratum.

^b^*P*-value for maternal genotype X alcohol interaction, assuming a linear trend for categories of alcohol drinking in 11st trimester.

^c^Referred to the mother. Carriers of the rare allele on average drank less alcohol.

Table 5 shows mean child IQ and KS2 scores by amount of alcohol the mothers reported drinking during the first trimester. Although KS2 score point estimates were consistently higher for carriers and differences increased for increasing alcohol consumption, there was no robust statistical evidence of genotype X alcohol interaction, assuming a linear trend for drinking categories. Results for IQ scores, based on smaller numbers, were all compatible with the null hypothesis and did not reveal any systematic differences.

In sensitivity analyses, children’s *ADH1B* genotype was not associated with KS2 score, even after adjusting for maternal *ADH1B* (Supplementary Table S2, available as Supplementary data at *IJE* online). Analyses excluding women reporting binge drinking or consuming 7+ units/week were largely compatible with the main Mendelian randomization effect estimates [mean difference [95% confidence interval: 1.9 (0.3, 3.4) and 0.6 (−2.6, 3.8), for child KS2 and IQ, respectively]. Further sensitivity analyses for number processing domains found similar associations between maternal genotype and KS2’s mathematics component but lack of evidence of an association with IQ’s arithmetic component, despite larger differences in mean scores (Supplementary Table S3, available as Supplementary data at *IJE* online), compared with the overall KS2 and IQ scores, respectively (Table 5). Empirical checks for the presence of selection bias in sensitivity analyses limited to the subsample with IQ data revealed point estimates for the genotype-KS2 associations similar to those from the main analyses, although the smaller sample size meant that there was little statistical evidence against the null hypothesis (Supplementary Table S4, available as Supplementary data at *IJE* online). Similarly, the observational analyses of self-reported alcohol consumption and KS2 scores restricted to pairs with IQ data showed point estimates close to the main analyses, but predictably larger statistical variation (Supplementary Table S4, available as Supplementary data at *IJE* online).

## Discussion

### Overall results

In the ALSPAC study, self-reported alcohol use before and during early pregnancy was positively associated with social advantage as well as with higher offspring IQ at age 8 years and better performance in standard school assessments (KS2) at age 11 years. These associations were attenuated on adjustment for measures of social position and other potential confounders but they remained relatively strong and substantial, particularly for pre-pregnancy drinking, possibly reflecting residual confounding. In contrast, the offspring of women whose genotype was related to a propensity to drink less before and during early pregnancy had higher KS2 scores. Results for child IQ scores, based on smaller numbers, were all null. Associations of the genotype with number processing domains of both scores were similar to results for the combined scores, suggesting that KS2 mathematics component could be responsible for the overall effect.

### Timing of exposure in observational analyses

Given the increasing interest in identifying sensitive periods for intrauterine exposures and some preliminary evidence of pre-conception effects of maternal drinking,^22^ the comparison of the associations of two self-reported alcohol exposure variables likely to reflect exposure early in pregnancy deserves further comments. Favourable associations with offspring cognitive outcomes were more clearly apparent in relation to self-reported moderate alcohol use before pregnancy, compared with self-reported first-trimester use. This exposure was also more clearly associated with measured confounders’ profiles likely to have a positive effect on offspring cognition, such as higher education and professional social class, followed by not smoking in pregnancy, higher vitamins/iron dietary intakes (reflective of healthier diets and lifestyles) and lower depression scores (Table 2). Therefore, it was probably more vulnerable to the effect of residual confounding. Self-reported alcohol use in the first trimester may also be prone to be influenced by social desirability bias leading to a general dilution of apparent associations. An alternative explanation is that alcohol use pre-pregnancy is a more valid measure of exposure during the critical period of early neuro-development and that such alcohol consumption does have beneficial effects. This seems highly unlikely given the discrepancy between findings of the observational analysis and the Mendelian randomization approach.

### Comparison with the published literature

Despite some recent results suggesting that most levels of prenatal alcohol exposure were not associated with detrimental consequences on cognitive/behavioural outcomes,^10–17^^,^^23^ a number of previous studies based on the ALSPAC cohort had already provided reason to question the apparent beneficial effect of moderate drinking in early pregnancy. A comparison of maternal and paternal frequency of alcohol consumption in association with offspring IQ showed a similar apparently beneficial effect of mothers and fathers drinking, suggesting this was due to family-level confounding, not a positive influence of regular intrauterine exposure. This methodology provides evidence as to whether associations are likely to be due to biological intrauterine effects rather than confounding by shared environmental or genetic factors.^35^^,^^36^ This was the case in a study showing that children of mothers who drank 1+ glass/day and binged during pregnancy had lower birthweights than those whose mothers abstained,^37^ and a comparison with paternal drinking concluded this observation was likely due to alcohol use by the mother.^38^ Another study of binge drinking during pregnancy in ALSPAC found increased mental health problems in offspring at the age of 7 years.^39^ Finally, our recent study showed that four foetal *ADH* variants were associated with reduced IQ at age 8 years, particularly in offspring exposed to moderate maternal drinking in pregnancy (1–6 units/wk), adding support to the notion that any alcohol exposure *in utero* could be damaging to the foetus.^20^

To our knowledge, this is only the second study to explicitly use Mendelian randomization to investigate the association between prenatal alcohol exposure and children’s cognitive/educational outcomes. In our previous paper,^20^ Mendelian randomization was applicable on the basis of the theoretical effect of variation in maternal and offspring ADH genes on (maternal and foetal) ethanol metabolism, resulting in differences in foetal exposure to ethanol. The prior for the present study is even more robust—here the *ADH1B* genotype chosen as instrument has been empirically validated and confirmed to be related to reactions following alcohol ingestion,^40–43^ to alcohol addiction^42^^,^^44–46^ and alcohol consumption,^42–44^^,^^47^ in several populations including ours of pregnant women.^24^ Therefore to our present knowledge this is the SNP with the strongest link to alcohol behaviour in the mothers, and the largest variation in ethanol reaching the foetus is likely to come from maternal behavioural differences rather than (more subtle) foetal metabolic differences. Results from these two studies are in broad agreement with each other; however they cannot be directly compared. The present study additionally examined the association with school results in the attempt to increase sample size, given that power was an issue here with such a rare variant used as instrument but not in the previous paper where the groups defined by offspring genotype score were adequately sized (all *n* >200). In Lewis *et al.*, the fact that offspring genotypes only affected IQ in exposed children lends strong support to the hypothesis that (any) prenatal alcohol exposure is detrimental to cognitive development;^20^ however no inference could be drawn from the direction of effect *per se* while we ignore how those genotypes affect prenatal alcohol exposure. On the other hand, the present result of better academic scores in children of mothers less likely to drink alcohol (including binge drinking) before and during early pregnancy because of their *ADH1B* genotype suggests a detrimental effect of intrauterine alcohol exposure because of what we know of the metabolic consequences of carrying the genotype. However, a clear picture did not emerge from genotype X alcohol interaction models, and we discuss possible reasons for this below.

In line with our results are findings from another type of quasi-experimental design, a Swedish natural experiment (a brief change in alcohol licensing policy) showing evidence for long-term detrimental effects of intrauterine alcohol exposure including lower educational attainment.^48^ Important evidence also comes from a Human Genome Epidemiology Network review reporting an increased risk of a number of adverse developmental outcomes in children related to maternal and foetal polymorphisms of the *ADH1B* gene.^49^ Findings of individual studies were conflicting, possibly due to relatively small sample sizes. However, the authors concluded that there was an increased risk of adverse outcomes related to maternal and possibly to foetal genotype at *ADH1B*, hypothesized to be due to an effect of this genotype on alcohol intake.^49^ The present study strengthens the initial evidence collated in the review, with reference to educational performance. However, in our study there was no independent association of offspring genotype with KS2 scores, suggesting that the association we observe reflects maternal alcohol intake or metabolism, rather than foetal metabolism.

### Strengths and limitations

Estimating the magnitude of the causal effect of maternal alcohol consumption during pregnancy on children’s cognitive/school performance requires formal instrumental variable analyses, and additional assumptions: about the exact definition of the exposure (timing, pattern, etc)—which would be arbitrary; and about the magnitude of the genotype-alcohol effect—for which no external data exist relative to the pregnancy period, and it is notoriously problematic to rely on internal data.^50^ Instead, we adopted the proof-of-principle approach of testing for the causal effect of (genetically driven) differences in the exposure on children’s outcomes, which is a valid test while requiring fewer assumptions. In this framework, we still rely on untestable assumptions (detailed in the methods section), for instance that the genotype is not related to unmeasured confounding.^18^^,^^19^ However, empirical checks found no association between genotype and a range of measured socioeconomically patterned confounders strongly associated with maternal drinking, confirming that here like in other instances genotypes display no more correlation with socioeconomic, lifestyle and physiological phenotypes than expected by chance.^51^ On these lines, sensitivity analyses restricted to the IQ sample indicate that genotype-outcome estimates are unlikely to be affected by selection bias due to loss to follow-up.

The use of a single genetic instrument, as opposed to multiple instruments that would allow checks for pleiotropy, constitutes a limitation, but it is the only viable approach since genome-wide association studies to date have not been successful in identifying any additional variants related to alcohol consumption in the general population. However, we believe that there are advantages of using this particular genetic instrument, namely its well-understood functional effects on the modifiable exposure and the fact that despite the numerous independent studies examining its association with various traits, still no evidence has surfaced that would suggest a possible independent pathway acting on markers of cognitive performance. For this reason, we believe that the unexpected presence of a genotypic effect for the children of women reporting abstention in the first trimester can be more plausibly explained, rather than by pleiotropy, by misclassification (of actual drinking behaviour in early pregnancy, as well as possible effects of drinking later in pregnancy but not early or before pregnancy recognition), and residual confounding / selection bias (re-introduced by stratification by alcohol drinking).

Due to the low prevalence of the SNP in our population, we need a sufficiently large sample to carry out a Mendelian randomization study. Lack of statistical power could account for failure to find evidence for genotype X alcohol interaction in models for KS2 score, for which a main effect was found, but numbers of rare allele carriers were too small for women drinking 1+ unit/week (*n* <20). Similarly, it is theoretically possible that the IQ sample could be too small (and therefore underpowered) to provide evidence of detrimental alcohol effects. If this was the case, and if cognitive ability solely explained the school results effect, we would still expect effect sizes for IQ to be larger than observed. Therefore, other mechanisms are likely to be key for KS2, and in particular behaviour (as previously suggested by studies showing maternal binge drinking increases offspring behavioural problems^17^ and hyperactivity/inattention^39^).

We have used women’s self-reported drinking before the ALSPAC pregnancy as a measure of their drinking before recognition of pregnancy. Such self-reports yielded levels of consumption which were higher than those during pregnancy, as many women do not realize they are pregnant and thus do not alter their drinking behaviour soon after conception,^52^, but lower than those based on a nationally representative sample of women of similar age, suggesting that some of the ALSPAC mothers might have cut down drinking in preparation for the pregnancy.^53^ The accuracy of self-reported alcohol consumption is questionable, and this may be more of an issue here, because of social pressures to stop or reduce drinking during pregnancy,^54^ although these were probably less intense in the UK in 1991–92 then they are currently. This type of reporting bias could overestimate the magnitude of alcohol-outcomes associations only if the outcomes were affected by bias in the same direction as the reporting of alcohol (i.e. if it were socially desirable to achieve lower KS2).^55^ This was not the case here.

Finally, the use of the *ADH1B* genotype to inform alcohol exposure differences cannot rule out entirely the importance of maternal drinking in the postnatal period, although we showed that the genotype is a better predictor of alcohol use prenatally than postnatally, and a previous South African study suggested that prenatal effects attributed to maternal *ADH1B* genotype were not markedly attenuated by controlling for postnatal consumption.^56^ To achieve a complete separation of postnatal effects, however, requires a different genetic instrument with proven specificity of the timing of effect (altering alcohol behaviour only during gestation), such as the offspring genetic score we successfully used in our recent paper —intrauterine exposure being the only plausible alcohol exposure for these children.^20^ Another alternative could be a more complicated design: for example one where some of the children exposed to alcohol *in utero* continue in the family environment whereas others are adopted away.

## Conclusion

Self-reported drinking in pregnancy was associated with better cognition and school results; however findings from a Mendelian randomization analysis more robust to residual confounding were compatible with no effect or a detrimental effect (on school performance). Offspring of women whose genotype was related to a propensity to drink less before and during early pregnancy, and to metabolize ethanol faster, were on average exposed to lower levels of alcohol prenatally and performed better in standard school tests than offspring exposed on average to more alcohol. These results are generalizable at least to other populations of European origin, but replication of the Mendelian randomization analysis is needed in order to strengthen the evidence in terms of statistical power (from a larger study or one with a higher prevalence of the variant) and provide assurance that the assumptions were not violated (by employing one or more genetic instruments in the same and different pathways).

The difference in alcohol levels reaching the foetus between the two groups defined by maternal genotype and corresponding to small but real differences in school results is likely to be very small, and for now impossible to quantify. This indicates once more that caution is needed with regard to any level of drinking during pregnancy, and advice to avoid alcohol also when attempting to get pregnant is warranted.

## Supplementary Data

Supplementary data is available at *IJE* online.

## Funding

This work was supported by the Wellcome Trust [grant number 083506]. The UK Medical Research Council (MRC), the Wellcome Trust [grant number WT092731] and the University of Bristol currently provide core support for ALSPAC. The design of questions on parental alcohol consumption was funded by a grant from the National Institute on Alcohol Abuse and Alcoholism to Dr Ruth Little. L.Z. was funded by an MRC Population Health Scientist fellowship [grant number G0902144]. L.Z. and G.D.S. work in a unit that receives funding from the UK MRC [G0600705] and the University of Bristol. Funding from the European Research Council grant DEVHEALTH [269874] is also gratefully acknowledged.

## Supplementary Material

Supplementary Data
