# Supplementary material for: Prenatal alcohol exposure and offspring cognition and school performance. A ‘Mendelian randomization’ natural experiment
Source: Int J Epidemiol. 2013 Sep 24;42(5):1358–70. doi: 10.1093/ije/dyt172 (PMC3807618; doi:10.1093/ije/dyt172)
Supplement: Supplementary Data [file supp_42_5_1358__index.html]

Prenatal alcohol exposure and offspring cognition and school performance. A ‘Mendelian randomization’ natural experiment — Supplementary Data 

# Prenatal alcohol exposure and offspring cognition and school performance. A ‘Mendelian randomization’ natural experiment

## Supplementary Data

files

**Files in this Data Supplement:**

- Supplementary Data - doc file
