# Supplementary material for: Prenatal alcohol exposure and offspring cognition and school performance. A ‘Mendelian randomization’ natural experiment
Source: Int J Epidemiol. 2013 Sep 24;42(5):1358–70. doi: 10.1093/ije/dyt172 (PMC3807618; doi:10.1093/ije/dyt172)
Supplement: Supplementary Data [file supp_dyt172_ije-2012-10-1039-File004.doc]

**Supplementary material (online only)**

Table S1. Association Between Alcohol Consumption Before, During Pregnancy (in First Trimester) and After Pregnancy (8 Months Post-Delivery), and the Rs1229984 Rare Allele (*ADH1B*) Among ALSPAC Mothers - Dominant Effect. Avon Longitudinal Study of Parents and Children, 1991–1992.

| Time period | Maternal alcohol consumption levels | N | Proportion carrying rare allele - % | Chi-square test for trend | *P* |
| --- | --- | --- | --- | --- | --- |
| Before pregnancy | 0 units/week | 487 | 6.5 | 13.15/1df | 0.0003 |
|  | < 1 unit/week | 2619 | 5.7 |  |  |
|  | 1-6 units/week | 3155 | 4.2 |  |  |
|  | 7+ units/week | 823 | 3.2 |  |  |
| First trimester | 0 units/week | 3164 | 5.8 | 14.81/1df | 0.0001 |
|  | < 1 unit/week | 2775 | 4.1 |  |  |
|  | 1-6 units/week | 1011 | 3.2 |  |  |
|  | 7+ units/week | 134 | 3.0 |  |  |
| Baby 8 months | 0 units/week | 774 | 5.6 | 5.43/1df | 0.02 |
|  | < 1 unit/week | 3056 | 5.3 |  |  |
|  | 1-6 units/week | 2388 | 3.9 |  |  |
|  | 7+ units/week | 209 | 4.3 |  |  |

Table S2. Estimates of the Mean Difference in Child’s KS2 Score at Age 11 by Mother and Child *ADH1B* Genotype in Different Models. Avon Longitudinal Study of Parents and Children, 1991–1992.

|  | Number | Mean | SE | *P* |
| --- | --- | --- | --- | --- |
| Mother *ADH1B* | 6268 | 1.77 | 0.66 | 0.009 |
| Mother *ADH1B* adjusted for child *ADH1B* ^a^ | 4971 | 2.26 | 0.86 | 0.009 |
| Child *ADH1B* | 7915 | 0.68 | 0.47 | 0.150 |
| Child *ADH1B* adjusted for mother *ADH1B** | 4971 | 0.22 | 0.83 | 0.792 |

SE – standard error

^a^ Test for interaction between mother and child *ADH1B*: *P*=0.491.

Table S3. Prenatal Alcohol Exposure and Number Processing - Arithmetic Component of WISC and Mathematics Component of KS2 Scores at Age 11 by *ADH1B*, Stratified by Maternal Alcohol Intake in 1^st^ Trimester. Avon Longitudinal Study of Parents and Children, 1991–1992. Models adjusted for ancestry-informative principal components to account for population stratification.

| Response | Alcohol  Drinking  in 1^st^ trimester | Numbers in analysis | | Effect estimate | | *P** | *P*** |
| --- | --- | --- | --- | --- | --- | --- | --- |
|  |  | Carrier ^a^ | Non-carrier ^a^ | Mean difference | 95% CI |  |  |
| ARITHM IQ score | 0 units/week | 76 | 1221 | -0.2 | -3.7, 3.3 | 0.917 |  |
|  | < 1 unit/week | 52 | 1207 | 0.4 | -3.7, 4.6 | 0.974 |  |
|  | 1-6 units/week | 10 | 439 | 1.2 | -7.8, 10.2 | 0.961 |  |
|  | 7+ units/week | 2 | 53 | 9.3 | -11.9, 30.5 | 0.686 |  |
|  | Overall | 140 | 2915 | 0.3 | -2.2, 2.8 | 0.820 |  |
|  |  |  |  |  |  |  | 0.504 |
| MATH KS2 score | 0 units/week | 113 | 1923 | 1.3 | -0.6, 3.2 | 0.177 |  |
|  | < 1 unit/week | 67 | 1755 | 1.5 | -0.9, 3.8 | 0.194 |  |
|  | 1-6 units/week | 17 | 623 | 1.4 | -3.3, 6.2 | 0.339 |  |
|  | 7+ units/week | 2 | 85 | 7.0 | -6.4, 20.5 | 0.238 |  |
|  | Overall | 199 | 4386 | 1.4 | 0.0, 2.8 | 0.046 |  |
|  |  |  |  |  |  |  | 0.670 |

|  |  |  | |  | |  |  |
| --- | --- | --- | --- | --- | --- | --- | --- |
|  |  |  |  |  |  |  |
|  |  |  |  |  |  |  |  |
|  |  |  |  |  |  |  |
|  |  |  |  |  |  |  |  |
|  |  |  |  |  |  |  |  |
|  |  |  |  |  |  |  |  |
|  |  |  |  |  |  |  |  |
|  |  |  |  |  |  |  |  |
|  |  |  |  |  |  |  |
|  |  |  |  |  |  |  |  |
|  |  |  |  |  |  |  |  |
|  |  |  |  |  |  |  |  |
|  |  |  |  |  |  |  |  |

MATH KS2 – Mathematics component of Key Stage 2; ARITHM IQ –Arithmetic component of WISC intelligent quotient; CI – confidence interval

* *P* values from t tests for differences of means within each drinking stratum.

** *P* value for maternal genotypeXalcohol interaction, assuming a linear trend for categories of alcohol drinking in 1^st^ trimester.

^a^ Referred to the mother. Carriers of the rare allele on average drank less alcohol.

Table S4. Sensitivity analysis KS2 scores effect estimates restricted to mother-child pairs with available IQ scores – Associations: a) with Alcohol Consumption Before and During Pregnancy (First trimester) and b) with ADH1B rare allele carrier status, Stratified by Maternal Alcohol Intake in First Trimester. Avon Longitudinal Study of Parents and Children, 1991–1992. Genetic Models adjusted for ancestry-informative principal components to account for population stratification.

a)

|  | Before pregnancy  Mean (SE) | | | First trimester  Mean (SE) | | |
| --- | --- | --- | --- | --- | --- | --- |
|  | N | Crude | Adjusted^a^ | N | Crude | Adjusted^a^ |
| 0 units/week | 302 | 0 | 0 | 2294 | 0 | 0 |
| < 1 unit/week | 2143 | 1.54 (0.51) | 0.65 (0.50) | 2349 | 0.34 (0.24) | 0.09 (0.23) |
| 1-6 units/week | 2580 | 2.89 (0.51) | 0.92 (0.50) | 774 | 0.85 (0.35) | 0.20 (0.34) |
| 7+ units/week | 686 | 3.91 (0.58) | 1.39 (0.58) | 89 | -0.09 (0.90) | -0.01 (0.91) |
| *P* ^b^ |  | <0.0001 | 0.010 |  | 0.030 | 0.591 |

b)

| Response | Alcohol  Drinking  in 1^st^ trimester | Numbers in analysis | | Effect estimate | | *P** | *P*** |
| --- | --- | --- | --- | --- | --- | --- | --- |
|  |  | Carrier ^a^ | Non-carrier ^a^ | Mean difference | 95% CI |  |  |
| KS2 score | 0 units/week | 69 | 1128 | 1.0 | -0.9, 3.0 | 0.300 |  |
|  | < 1 unit/week | 48 | 1102 | 0.5 | -1.8, 2.9 | 0.526 |  |
|  | 1-6 units/week | 10 | 396 | 1.8 | -2.5, 6.1 | 0.421 |  |
|  | 7+ units/week | 2 | 49 | 6.2 | -5.3, 17.7 | 0.334 |  |
|  | Overall | 129 | 2675 | 1.0 | -0.4, 2.4 | 0.157 |  |
|  |  |  |  |  |  |  | 0.793 |

KS2 – Key Stage 2; IQ – intelligent quotient; SE – standard error

^a^ Adjusted for family social class and the following maternal characteristics: age, education, parity, smoking during pregnancy, diet (calcium, vitamin C, iron and folate intake), Edinburgh postnatal depression score.

^b^ P-values for linear trend across categories of alcohol consumption
